# Cytotoxicity of eupatorin in MCF-7 and MDA-MB-231 human breast cancer cells via cell cycle arrest, anti-angiogenesis and induction of apoptosis

**DOI:** 10.1038/s41598-018-37796-w

**Published:** 2019-02-06

**Authors:** Nursyamirah Abd Razak, Nadiah Abu, Wan Yong Ho, Nur Rizi Zamberi, Sheau Wei Tan, Noorjahan Banu Alitheen, Kamariah Long, Swee Keong Yeap

**Affiliations:** 1 Laborotary of Vaccines and Immunotherapeutics, Institute of Bioscience, Universiti Putra Malaysia, Serdang, 43400 Selangor Malaysia; 2 UKM Molecular Biology Institute (UMBI), UKM Medical Centre, Jalan Yaa’cob Latiff, Bandar Tun Razak, Cheras, 56000 Kuala Lumpur Malaysia; 3 School of Biomedical Sciences, The University of Nottingham Malaysia Campus, Jalan Broga, Semenyih, 43500 Selangor Malaysia; 4 Department of Cell and Molecular Biology, Faculty of Biotechnology and Biomolecular Sciences, Universiti Putra Malaysia, Serdang, 43400 Selangor Malaysia; 5 Malaysian Agricultural Research and Development Institute (MARDI), Serdang, 43400 Selangor Malaysia; 6 China-ASEAN College of Marine Sciences, Xiamen University Malaysia, Jalan Sunsuria, Bandar Sunsuria, Sepang, 43900 Selangor Malaysia

## Abstract

Eupatorin has been reported with *in vitro* cytotoxic effect on several human cancer cells. However, reports on the mode of action and detail mechanism of eupatorin *in vitro* in breast cancer disease are limited. Hence, eupatorin’s effect on the human breast carcinoma cell line MCF-7 and MDA-MB-231 was investigated. MTT assay showed that eupatorin had cytotoxic effects on MCF-7 and MDA-MB-231 cells but was non-toxic to the normal cells of MCF-10a in a time-dose dependent manner. At 24 h, the eupatorin showed mild cytotoxicity on both MCF-7 and MDA-MB-231 cells with IC_50_ values higher than 20 μg/mL. After 48 h, eupatorin at 5 μg/mL inhibited the proliferation of MCF-7 and MDA-MB-231 cells by 50% while the IC_50_ of MCF-10a was significantly (p < 0.05) high with 30 μg/mL. The concentration of eupatorin at 5 μg/mL induced apoptosis mainly through intrinsic pathway by facilitating higher fold of caspase 9 compared to caspase 8 at 48 h. The cell cycle profile also showed that eupatorin (5 μg/mL) exerted anti-proliferation activity with the cell cycle arrest of MCF-7 and MDA-MB-231 cells at sub Gθ/G1 in a time-dependent manner. In addition, wound healing assay showed an incomplete wound closure of scratched MDA-MB-231 cells, and more than 60% of the MDA-MB-231 cells were prevented to migrate and invade the membrane in the Boyden chamber after 24 h. Eupatorin also inhibited angiogenic sprouting of new blood vessels in *ex vivo* mouse aorta ring assay. In gene expression assay, eupatorin up-regulated pro-apoptotic genes such as Bak1, HIF1A, Bax, Bad, cytochrome c and SMAC/Diablo and blocked the Phospho-Akt pathway. In conclusion, eupatorin is a potent candidate to induce apoptosis and concurrently inhibit the invasion, migration and angiogenesis of MDA-MB-231 and MCF-7 cells through inhibition of Phospho-Akt pathway and cell cycle blockade.

## Introduction

Breast cancer is the most common form of cancer present in women worldwide and is the second leading cause of death after lung cancer^1,2^. Among all breast cancer types, triple negative breast cancer (TNBC) is the most aggressive; it is difficult to treat and more likely to spread in diagnosed patients. Women with TNBC have poor prognosis with few treatment options; therefore, new therapeutic agents for this aggressive tumour are critically needed^3^.

Numerous researchers found that flavonoids are capable to inhibit cancer cell proliferation and delay tumour progression^4,5^ via supressing the metastasis, angiogenesis^6^ and by regulating many apoptosis related signaling pathways such as Akt and PTEN pathways^7,8^. Therefore, consumption of food containing flavonoids may help to prevent the initiation or early progression of cancer cells in cancer patients. Eupatorin (3′,5-dihydroxy-4′,6,7-trimethoxyflavone) is one of the potent candidates as anti-breast cancer agents^9,10^. This bioactive compound belongs to the flavone group, commonly found in a variety of fruits, vegetables, and herbs^6^. Previous research reported that eupatorin potently suppresses proliferation and induces apoptosis in multiple cancer cell lines^10,11^. However, the detailed efficacy and mechanisms of eupatorin as anti-breast cancer agent *in vitro* are very limited.

In most breast cancer cases, the expression level of ERα is directly proportional to tumour growth^12^. Therefore, the MCF-7 cell model has been examined extensively to determine the mechanism of estrogen-stimulated growth in tumour^13^. In addition, MDA-MB-231 (estrogen-receptor negative) cells that are aggressive and invasive triple negative breast cancer (TNBC) cells are known to be resistant to several anti-cancer agents^14^. Hence, this study was aimed to evaluate the cytotoxic effect and apoptosis induction of eupatorin in MCF-7 and MDA-MB-231 cells line *in vitro*. For comparison, antiproliferative effects of eupatorin on MCF-10a human breast epithelial cells were also examined. By comparing breast cancer cell lines (MCF-7 and MDA-MB-231) with MCF-10a, the selectivity of eupatorin cytotoxic effects on cancer cells was determined.

## Results

### Eupatorin inhibited proliferation of human breast adenocarcinoma MCF-7 and MDA-MB-231 cells

Cytotoxic effect of eupatorin on MCF-7 and MDA-MB-231 cells was evaluated using MTT assay. As shown in Supplementary Fig. 1(A–C), eupatorin caused a time (24, 48 and 72 h) and dosage (0.16–20 μg/mL) dependent inhibition of cell proliferation towards MCF-7 and MDA-MB-231 (Table 1). At 24 h, the IC_50_ value of eupatorin was higher than 20 μg/mL for both cell types. When the incubation was extended for 48 h, the MCF-7 and MDA-MB-231 cells exhibited the IC_50_ value of 5 μg/mL. At 72 h, the IC_50_ of MCF-7 and MDA-MB-231 cells was 3 μg/mL and 2 μg/mL, respectively. In contrast, eupatorin was recorded with much lower cytotoxic effect to MCF-10a cells, which exhibited high IC_50_ value of 30 μg/mL at 72 h.

**Table 1:** IC_50_ value and selective index of eupatorin (after 24, 48 and 72 hour incubation time) and positive control doxorubicin (after 48 hour incubation time) on MCF-10a, MCF-7, and MDA-MB-231 cells.

Cell line	IC_50_ value at different time point (μg/mL)			
	Eupatorin			Doxorubicin
	24 hr	48 hr	72 hr	48 hr
MCF-10a	>30	30.00 ± 0.01	30.00 ± 0.02	0.48 ± 0.33
MCF-7	>20	5.00 ± 0.01	3.00 ± 0.02	0.52 ± 0.26
MDA-MB-231	>20	5.00 ± 0.07	2.00 ± 0.05	0.71 ± 0.21
Selective index (SI) MCF-10a/MCF-7	—	6	10	0.92
Selective index (SI) MCF-10a/MDA-MB-231	—	6	15	0.68

Values are expressed as mean ± S.D for three independent observations.

The selectivity Index (SI) in Table 1 shows that cytotoxic effects of eupatorin were selected towards MCF-7 and MDA-MB-231 cells where the SI value of 6 was recorded for respective cells type at 48 h, respectively. Interestingly, these values were 6.5 and 8.8 times higher than the positive control doxorubicin. Subsequently, the IC_50_ value of eupatorin at 48 h (5 μg/ml) was selected to treat MCF-7 and MDA-MB-231 cells in further experiments.

### Morphological assessment

#### Phase contrast microscopy

**Figure 1:**
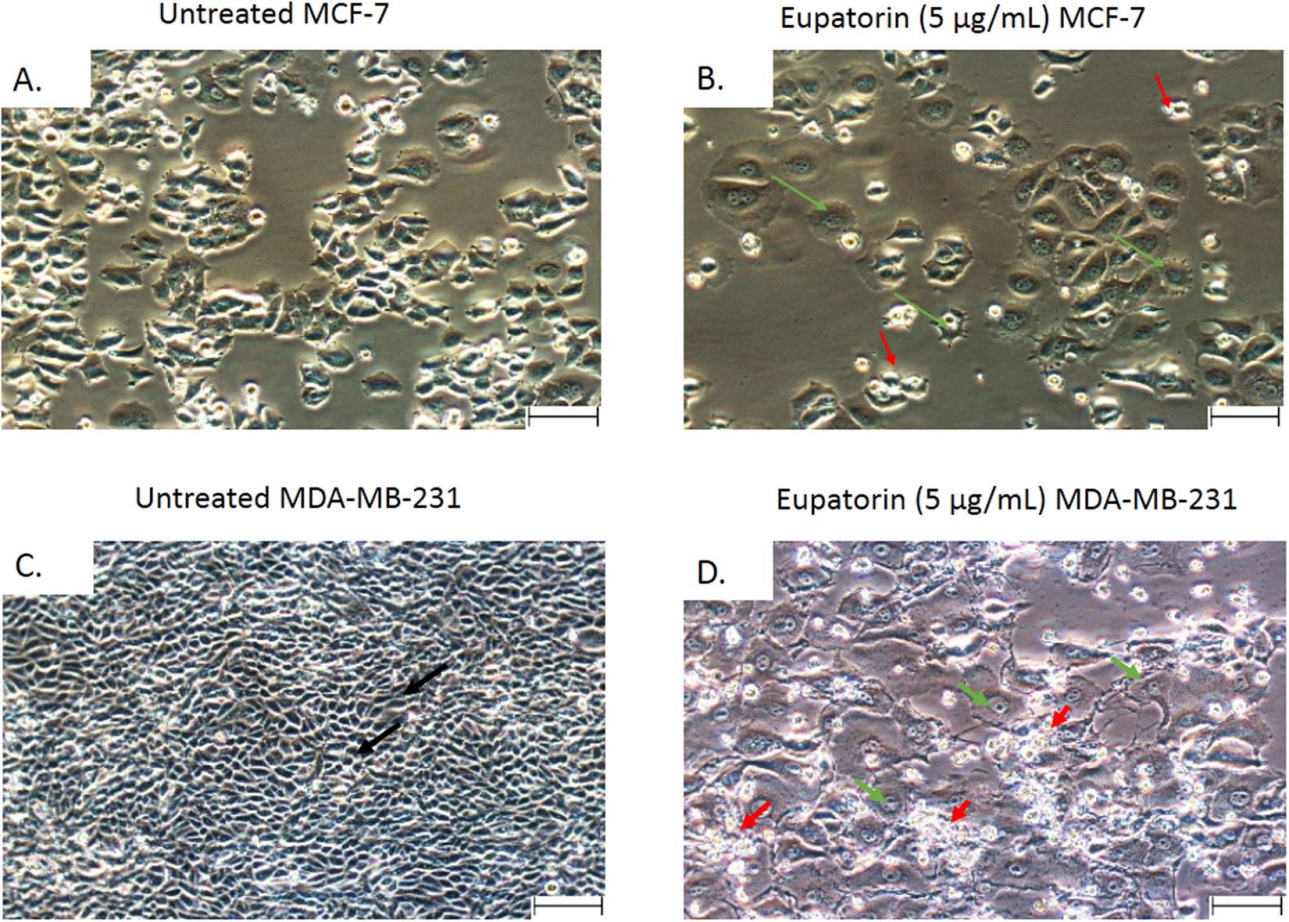
Representative photomicrographs of the cellular morphology of (**A**) untreated MCF-7 and (**B**) eupatorin (5 µg/mL) treated MCF-7 (**C**) untreated MDA-MB-231 and (**D**) eupatorin (5 µg/mL) after 72 hours incubation. Cells were viewed using Nikon (Japan) microscope. Green arrow () show cells became rounded and shrunken while red arrow () pointed at the suspension of dead cells. Magnification: x40; scale bar: 50 μM.

As shown in Fig. 1A–C, the untreated MCF-7 and MDA-MB-231 cells maintained their original morphology and close contact to each other even when the incubation was prolonged to 72 h. In contrast, the MCF-7 and MDA-MB-231 cells lost their original shape at 24 h of eupatorin treatment. The MCF-7 cells were not in their polygonal or trigonal shape while MDA-MB-231 cells had lost their elongated spindle-shape morphology. When the treatment was extended to 48 h, suspension cells (dead cells) were identified and more suspension cells were observed at 72 h (Fig. 1B,D).

#### Morphological changes of MCF-7 and MDA-MB-231 cells apoptosis by SEM

**Figure 2:**
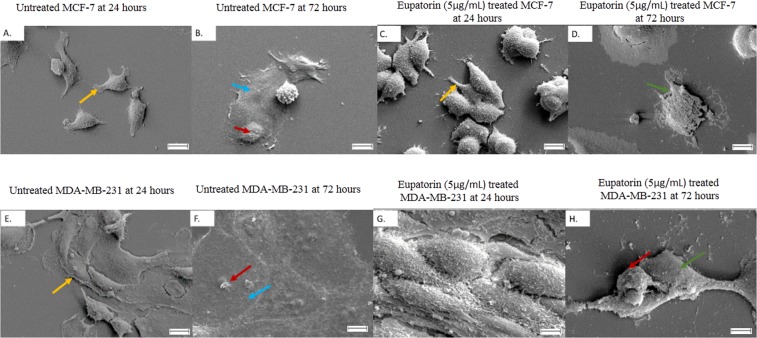
Morphology changes in MCF-7 and MDA-MB-231 cells after eupatorin treatment at 72 hours were recorded using SEM. (**A**) untreated MCF-7 at 24 hours, (**B**) untreated MCF-7 at 72 hours, (**C**) eupatorin-treated MCF-7 cells at 24 hours and (**D**) eupatorin-treated MCF-7 cells (**E**) untreated MDA-MB-231 at 24 hours, (**F**) untreated MDA-MB-231 at 72 hours, (**G**) eupatorin-treated MDA-MB-231 cells at 24 hours and (**H**) eupatorin-treated MDA-MB-231 cells at 72 hours. Blue arrow () indicated the membrane collapsed, green arrow () indicated complete loss of cells membrane, and red arrow () indicated shrunken cell and the absence of microvilli while yellow arrow () pointed at intact body of healthy cells. Magnification: x1000; scale bar: 10 μM.

Through SEM, morphological changes in MCF-7 and MDA-MB-231 cells were characterized in detail. The untreated MCF-7 cells (Fig. 2A,B) showed their intact membrane with its pseudopodia were at full stretch, tightly sticking on the cover glass while the untreated MDA-MB-231 cells (Fig. 2E,F) exhibited abundant microvilli and cellular crowding with cytoplasmic connection, which suggests healthy proliferation. However, morphologically damage cells were observed in the eupatorin treated MCF-7 and MDA-MB-231 groups at 24 h (Fig. 2C,G). When the treatment was extended to 72 h, MCF-7 cells were clearly shrunk to abnormal round (Fig. 2D) while MDA-MB-231 cells were shrunk and lost their microvilli (Fig. 2H).

#### Eupatorin inhibited the migration and invasion of MDA-MB-231 cells

**Figure 3:**
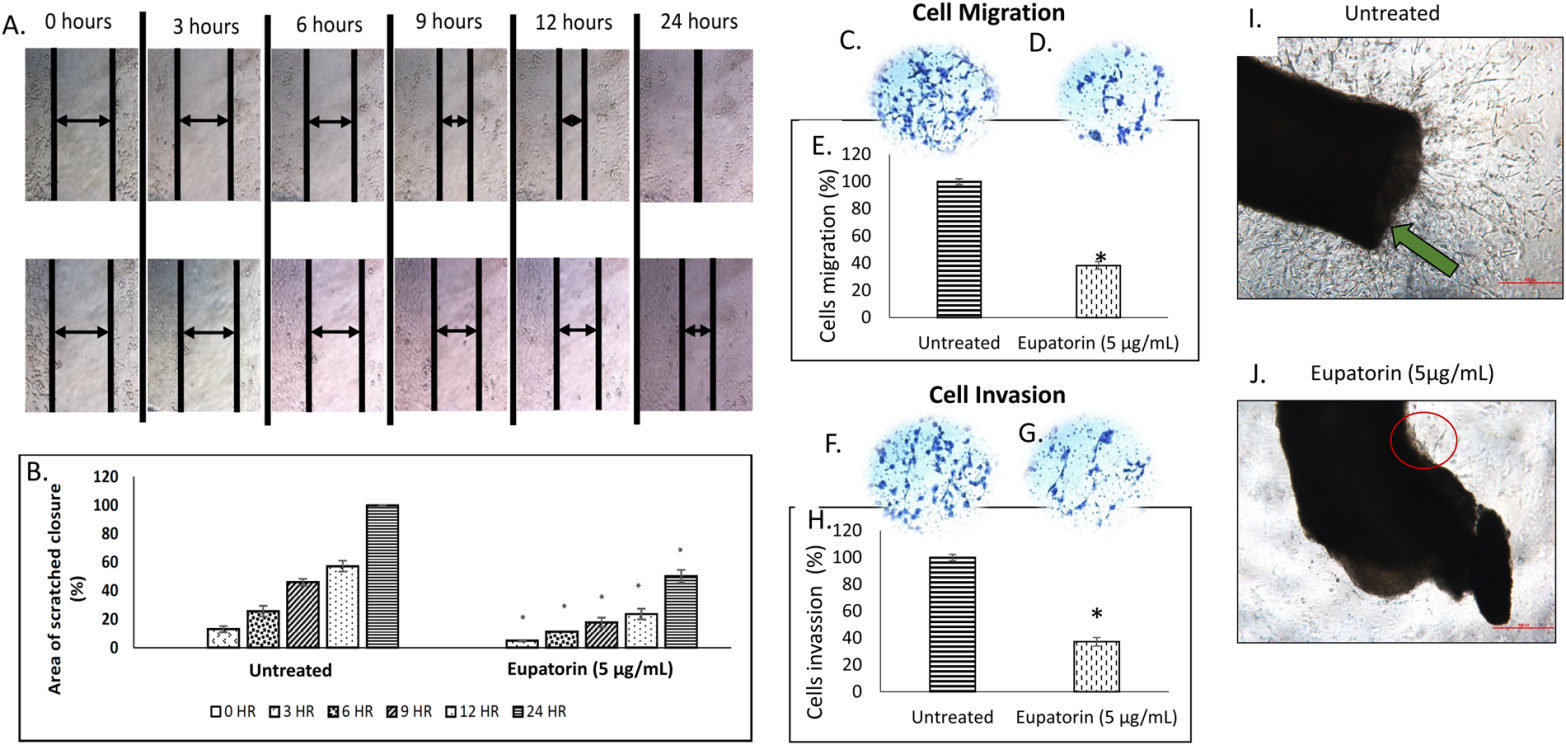
(**A**) Representative image and (**B**) quantitative closure (%) for scratched assay. The confluent MDA-MB-231 cells were incubated with 5 μg/mL for the indicated times. The widths of injury lines made in cells were then examined at 0, 3, 6, 9, 12 and 24 hours. Images were taken using microscope (Nikon, Japan) at indicated periods. Magnification: x10; scale bar: 50 μm. Eupatorin inhibits serum-induced MDA-MB-231 cell (**C–E**) migration and (**F,G**) invasion in Boyden chamber assay after 24 hours. The pictures (**C,D,F,G**) show a section of the visual field of one representative experiment (x10 magnification). Scale bar: 50 μm. (**C**) migration of untreated MDA-MB-231 cells through membrane in the Boyden chamber assay, (**D**) migration of treated MDA-MB-231 cells, (**E**) percentage of migrated MDA-MB-231 cells, (**F**) invasion of untreated MDA-MB-231 cells, (**G**) invasion of treated MDA-MB-231 cells and (**H**) percentage of invaded MDA-MB-231 cells through Matrigel-coated membrane in the Boyden chamber assay. Statistical analysis was performed using unpaired t-test. (**I**) Untreated mouse aorta ring (**J**) eupatorin treated mouse aorta ring. Eupatorin inhibited the formation of new blood vessels in mouse aorta after 10 days incubation. Mouse aorta ring was viewed using Nikon microscope at x40 magnification. Scale bar: 100 μm. Green arrow indicated the formation of massive new blood vessels on aortic ring. Data are presented as mean values ± SD of n = 10 independent experiments. (^*^statistical significance (p < 0.05) against the untreated group).

The effect of eupatorin on cell migration was examined using the scratch assay (Fig. 3A,B). As shown in Fig. 3B, eupatorin (5 μg/mL) significantly (p < 0.05) decreased cell motility and migration of cancer cells where the complete closure of the scratched area was inhibited by 50.14% at 24 h. In addition, Transwell Boyden chamber assay showed that eupatorin prevented MDA-MB-231 cells from migrating aggressively through the filter (Fig. 3C,D). Only 38 ± 3% of MDA-MB-231 cells migrated through the membrane compared to the untreated group (Fig. 3E). In addition, Fig. 3F,G show that eupatorin significantly (p < 0.05) decreased the level of cell invasion through the Matrigel-coated membrane. Only 37 ± 3% of MDA-MB-231 cells had invaded the Matrigel-coated membrane in the Boyden chamber assay (Fig. 3H) at 24 h. Taken together, these results suggest that eupatorin has strong inhibitory effects on the migration and invasion of MDA-MB-231 cells.

#### Effects of eupatorin in angiogenic inhibition

To study the role played by eupatorin in angiogenesis, mouse aortic rings assay was carried out using aorta isolated from Balb/c mice (Fig. 3I,J). As shown in Fig. 3J, mouse aorta ring of Balb/c mouse treated with eupatorin (5 μg/mL) exhibited a small amount of vascular sprouting of new blood vessels from the aorta as pointed out in red circle. In contrast, the green arrow pointed out the massive tube formation around the tissue section of the untreated mouse aorta (Fig. 3I). Sprouts from the control mouse aorta rings were longer in length and more branches were formed. Therefore, *ex vivo* model using aortic ring from Balb/c mouse suggests that eupatorin can act as an anti-angiogenic agent.

#### Effect of eupatorin on the cell cycle distribution in MCF-7 and MDA-MB-231 cells

**Figure 4:**
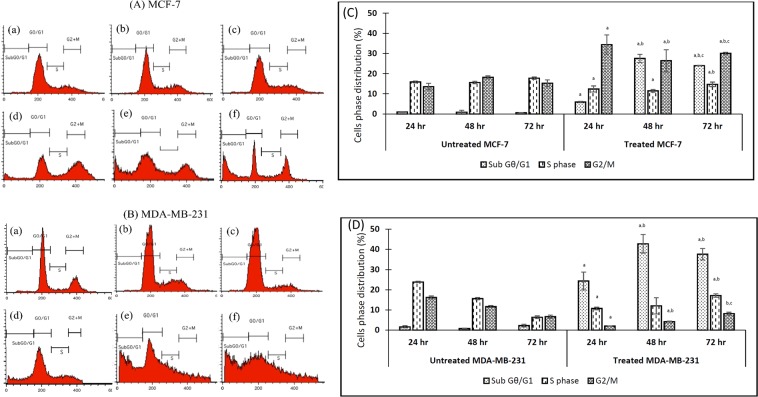
The regulatory effect of eupatorin on cell cycle distribution in (**A**) MCF-7 and (**B**) MDA-MB-231 cells. The cells were treated with specific concentration of eupatorin for 24, 48 and 72 h. (**A**) Flow cytometry assay of eupatorin-induced apoptosis cells. (a) untreated for 24 h; (b) untreated for 48 h; (c) untreated for 72 h; (d) treatment with 5 μg/mL eupatorin for 24 h; (e) treatment with 5 μg/mL eupatorin for 48 h; (f) treatment with 5 μg/mL eupatorin for 72 h. (C and D) Columns show mean values of three experiments (±S.D.). (**C**) eupatorin inhibited cell cycle progression in MCF-7 and (D) eupatorin inhibited cell cycle progression in MDA-MB-231 cells. (^a^Statistical significance (p < 0.05) compared to the untreated for the respective time point, ^b^statistical significance (p < 0.05) compared to the treated group for 24 hours, ^c^statistical significance (p < 0.05) compared to the treated group for 48 hours).

The cell cycle analysis for control and treated MCF-7 (Fig. 4A) and MDA-MB-231 (Fig. 4B) was analyzed using a flow cytometer. The results showed that 34.40% ± 4.7 MCF-7 cells that were exposed to eupatorin for 24 h were arrested in the G2/M phase while 12.37% ± 1.51 of treated cells were distributed in S phase (Fig. 4C). In addition, a small percentage of MCF-7 cells (5.89% ± 0.30) were in sub Gθ/G1 transition. On the other hand, Fig. 4D shows that 24.33% ± 4.37 of MDA-MB-231 cells were accumulated in the sub Gθ/G1 phase while cells in G2/M phase and S phase was 2.00% ± 0.09 and 10.73% ± 0.61 respectively. At 48 h treatment, the number of MCF-7 cells accumulated in sub Gθ/G1 was extremely increased to 27.52% ± 2.06 while cell in G2/M phase was 26.41% ± 5.48 whereas the number of MDA-MB-231 cells accumulated in sub Gθ/G1 was remarkably high which exhibited 42.75% ± 4.67. When the treatment was prolonged to 72 h, the number of MCF-7 cells arrested in G2/M phase was 30.06% ± 0.56 while cells accumulated in sub Gθ/G1 slightly decreased to 23.99% ± 0.13. For MDA-MB-231 cells, the cells percentage in sub Gθ/G1 was slightly decreased to 37.54% ± 2.82. However, the number of cells arrested in S phase was increased to 17.13% ± 0.88. These data showed that eupatorin can act both as apoptosis inducer and cells growth inhibitor in MCF-7 and MDA-MB-231 cells in a time dependent manner.

#### Eupatorin induced apoptosis in MCF-7 and MDA-MB-231 cells line

**Figure 5:**
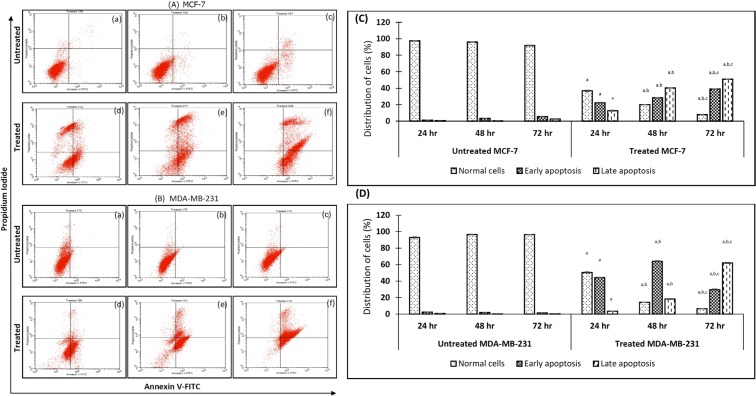
Eupatorin-induced apoptosis in MCF-7 and MDA-MB-231 cells by AnnexinV-FITC/PI staining assay. The cells were treated with specific concentration of eupatorin for 24, 48 and 72 h. (**A**) Flow cytometry assay of eupatorin-induced apoptosis cells. (a) untreated for 24 h; (b) untreated for 48 h; (c) untreated for 72 h; (d) treatment with 5 μg/mL eupatorin for 24 h; (e) treatment with 5 μg/mL eupatorin for 48 h; (f) treatment with 5 μg/mL eupatorin for 72 h. (C and D) Columns show mean values of three experiments (±S.D.). (**C**) eupatorin induced apoptosis of MCF-7 and (**D**) eupatorin induced apoptosis in MDA-MB-231 cells. (^a^Statistical significance (p < 0.05) compared to the untreated for the respective time point, ^b^statistical significance (p < 0.05) compared to the treated group for 24 hours, ^c^statistical significance (p < 0.05) compared to the treated group for 48 hours).

In this experiment, the effect of eupatorin on apoptosis induction in MCF-7 (Fig. 5A) and MDA-MB-231 (Fig. 5B) cells were examined. Dot-plot graphs illustrated the viable cells (the lower left quadrant), early-phase apoptotic cells (the lower right quadrant), late-phase apoptotic or dead cells (the upper right quadrant) and the necrotic cells (the upper left quadrant). As shown in Fig. 5C,D, the early apoptotic cell populations of MCF-7 and MDA-MB-231 in the lower right quadrant at 24 hours were 22.21% ± 0.52 and 44.33% ± 0.45, respectively. In addition, the percentage of late apoptotic cells for MCF-7 and MDA-MB-231 in the upper right quadrant were 12.73% ± 0.54 and 3.50% ± 0.20 respectively. At 48 h, eupatorin raised the respective fraction of early apoptotic and late apoptotic cells of MDA-MB-231 to 64.04% ± 0.66 and 18.27% ± 0.53. For the MCF-7 cells, eupatorin increased the population of early apoptotic and late apoptotic to 28.28% ± 0.24 and 40.26% ± 0.33, respectively. When the treatment was prolonged to 72 h, more than 50% of MCF-7 and MDA-MB-231 cells were in late apoptotic phase, the percentage of which was 50.85% ± 0.14 and 66.02% ± 0.57, respectively.

#### Effect of eupatorin on marker genes

**Figure 6:**
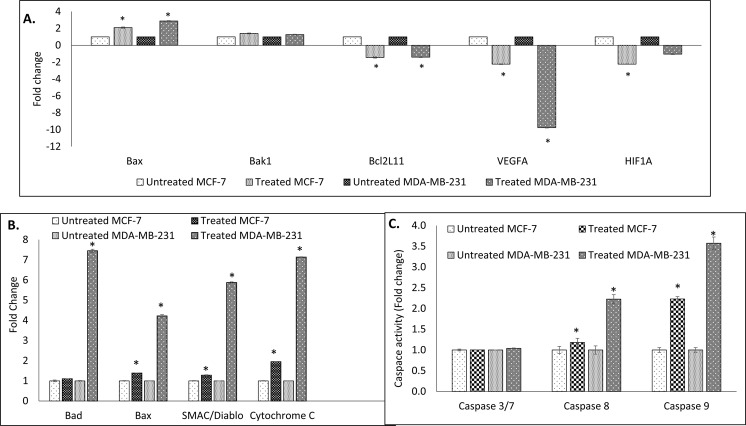
(**A**) Expression of Bax, Bak1, Bcl2L11, VEGFA and HIF1A genes in MCF-7 and MDA-MB-231 cells using real-time PCR (qPCR) machine. (**B**) Proteome profiling of (**A**) MCF-7 and (**B**) MDA-MB-231 cells after treated with 5 μg/mL eupatorin for 48 hours. (**C**) Activation of caspase 3/7, 8 and 9 activity in MDA-MB-231 and MCF-7 cells after 48 hours treatment with 5 μg/mL eupatorin. Statistical analysis was performed using unpaired t-test of one-way ANOVA. (^*^statistical significance (p < 0.05) against the untreated group).

As expected, the differences in genes expression were observed when cancer cells of MCF-7 and MDA-MB-231 cells were treated with eupatorin. According to Fig. 6A, the Bax gene was up-regulated while Bcl2L11, VEGFA and HIF1A genes were down-regulated in treated MCF-7 cells. In addition, eupatorin significantly (p < 0.05) promoted the expression of Bax and suppressed the Bcl2L11 and VEGFA expression in MDA-MB-231 cells compared to the untreated. However, there was no significant (p < 0.05) difference in Bak1 gene expressed in both types of cancer cells MCF-7 and MDA-MB-231 cells.

#### Eupatorin regulated the pro-apoptotic protein in MCF-7 and MDA-MB-231 cells

Proteome profiling assay had highlighted the involvement of several apoptotic proteins in promoting apoptosis in treated MCF-7 and MDA-MB-231 cells line (Fig. 6B) such as Bad, Bax, SMAC/Diablo and cytochrome c. In MCF-7, eupatorin regulated the pro-apoptotic proteins Bad and Bax with 1.10 ± 0.02 and 1.38 ± 0.01 fold respectively, while in MDA-MB-231 cells proteins Bad and Bax showed a changed value of 7.45 and 4.22 respectively.

#### Caspase activity in eupatorin-treated MCF-7 and MDA-MB-231 cells

Figure 6C demonstrates the activation of caspases activity including caspase 3/7, 8 and 9 in MCF-7 and MDA-MB-231 cells at 48 h treatment with eupatorin. Based on this figure, activation of caspase 9 was higher than caspase 8 in both types of cancer cells. Caspase 9 possessed 1.29 ± 0.02 fold while caspase 8 exhibited 1.11 ± 0.06 fold in MCF-7 cells. In MDA-MB-231 cells, caspase 8 and 9 were up-regulated to 2.23 ± 0.11 fold and 3.57 ± 0.15, respectively.

#### Eupatorin altered the expression in cell cycle/checkpoint

**Figure 7:**
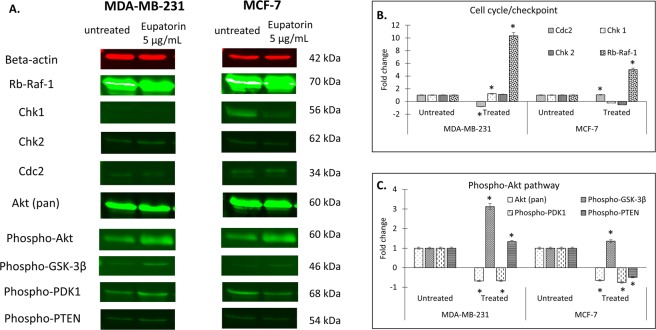
(**A**) Representative of stained-free gel for MDA-MB-231 and MCF-7 cells. The sample sequence are as follow: untreated cells (left) and cells treated with 5 μg/mL eupatorin (right). Eupatorin effected the targeted protein expression in (**B**) cell cycle/checkpoint and (**C**) Phospho-Akt pathway in MCF-7 and MDA-MB-231 cells line after 48 hours treatment.

In this study, regulation of protein expression in cell cycle for MDA-MB-231 and MCF-7 cells such as beta-actin (housekeeping protein), retinoblastoma (Rb-Raf-1), checkpoint kinase 1 (Chk1) and checkpoint kinase 2 (Chk2) was analyzed. The protein band is shown in Fig. 7A.

Based on Fig. 7B, eupatorin altered the protein levels in MCF-7 and MDA-MB-231 cells. The levels of Cdc2 molecules in MDA-MB-231 cells were reduced to 0.971 fold by eupatorin at dose of 5 μg/mL for 48 hours. In contrast, eupatorin enhanced the regulation of the expression of Chk1 (1.473), Chk2 (1.352) and Rb-Raf-1 (12.575), which belong to cell cycle stimulating molecules. Meanwhile, the protein expression of Chk1 and Chk2 in MCF-7 cells was suppressed by 5 μg/mL eupatorin at 48 hours incubation. In contrast, the protein of Cdc2 and Rb-Raf-1 in MCF-7 cells were enhanced by 1.290 and 6.123 fold at 48 hours of eupatorin treatment. Therefore, these results indicated that eupatorin may inhibit cells proliferation through depletion of Cdc2 protein in MDA-MB-231 and dysregulation of Chk1 protein level in MCF-7 cell cycle checkpoint to weaken invasion and migration in breast cancer cells through angiogenesis.

#### Eupatorin altered the protein expression in Akt pathway

In Akt pathway, alteration of protein level such as Akt (pan), Phospho-Akt, Phospho-GSK-3β, Phospho-PDK1, Phospho-PTEN in MDA-MB-231 and MCF-7 cells treated with 5 μg/mL eupatorin at 48 h was observed (Fig. 7C). The protein level of Phospho-GSK-3β in MDA-MB-231 cells was highly expressed with 3.901 fold followed by expression of phosphor-PTEN protein level (1.662 fold). However, Akt (pan) and Phospho-PDK1 were reduced by 0.842 and 0.832 fold, respectively. For MCF-7 cells, eupatorin increased the protein level of Phospho-GSK-3β (1.196 fold). In contrast, the protein level of Akt (pan), Phospho-PDK1 and Phospho-PTEN was suppressed by 0.570, 0.660 and 0.433 fold respectively in MCF-7 cells.

## Discussion

Eupatorin is a methoxyflavone, a group of flavonoid that shows great potential as cancer chemopreventive agents in cell culture studies^11,15^. It is known as an anti-proliferation and anti-mitotic flavonoid^10^. To be a potent anti-cancer in biological system, a flavone should consist of methoxy groups that metabolize faster to the conversion products than their hydroxylated analogues^16^. Previously, the anti-proliferative effects of eupatorin were proposed as a result of CYP1 family (cytochrome P450 CYP1) enzyme metabolism^9^. CYP1A1, CYP1A2 and CYP1B1 are the members of human CYP1 family enzymes that participated in phase I metabolism of drugs in the liver^17,18^. Overexpression of CYP1A1 and CYP1B1 were reported in various types of cancer cells including breast cancer^19^. Eupatorin, which possesses the structural features (being planar, neutral, and aromatic), makes it fit in well within CYP1A2 cavity^20^. Other studies have documented that besides CYP1A2, eupatorin also can be metabolized by CYP1A1 and CYP1B1. These CYP1 families promoted hydroxylation in eupatorin at 4′ position in the B ring with the presence of neighboring pre-existing substitution that were not hydroxylated to form flavone cirsiliol^9,16^. The bioconversion of eupatorin to more active product such as cirsiliol enhanced the antiproliferative activity. Further hydroxylation by CYP1 family enzymes may occur within cirsiliol at 5′, 6′ and 8′ positions in A ring and at 5′ position in B ring, which may enhance the antiproliferative activity^16^. Importantly, CYP1 family enzymes were not expressed in MCF-10a cells^9^. Thus, eupatorin at 5 μg/mL did not affect the viability of human normal breast MCF-10a cells and was found highly selective towards cancer cells indicated by SI>3^21,22^. Interestingly, although the IC_50_ value of eupatorin was far higher than the common anticancer drug doxorubicin on both cancer cell lines, the MCF-10a vs MCF7 and MCF-10a vs MDA-MB-231 SI values of eupatorin were 6.5 and 8.8 times higher than doxorubicin, respectively. This result had shown the advantage and potential of further exploring the potential antitumor effect of eupatorin.

Exposure of eupatorin to the pre-mitotic cells also caused defects in spindle structure and centrosome function that resulted in a mitotic delay in anaphase^10^. This triggered a transient M phase and potently induced apoptosis in multiple cancer cell lines that suppressed cancer cell proliferation^10^. During eupatorin treatment, breast cancer cells lost their epithelial properties including cell–cell contacts, degradation of microvilli and destruction of membrane cells. In the tumor microenvironment, elevated levels of MMPs such as MMP-9 were able to induce epithelial-mesenchymal-transition (EMT), whereas EMT itself promotes MMP-9 secretion that facilitates cell invasion and metastasis^23^. EMT is a cell process by which epithelial cells converse into mesenchymal cells and obtain the capacity of cell motility to maintain the invasion and metastasis of malignancies^24^. Hence, the knockdown of MMP-9 leads to down-regulation of EMT that inhibites the migration and invasion of aggressive breast cancer cells. Gene MMP-9 and VEGFA play a critical and dual role in cancer metastasis and angiogenesis to foster progression of breast cancer cells *in vitro*^25,26^. Previous studies reported that gene of MMP9 is essential for cell migration^26^ while VEGFA is a member of the family of platelet-derived growth factors, which is one of the primary pro-angiogenic factors that encourage the formation of new blood vessels in the tumour microenvironment^27–32^. Thus, down-regulation of either MMP9 or VEGFA expressions could further suppress the metastatic potential and angiogenic of cancer cells^33^.

Reduction of MMP9 expression is commonly associated with the increase of mitochondrial membrane permeability, which facilitates the release of pro-apoptotic factors^34^. The activation of protein Bax, Bad, cytochrome c and SMAC/Diablo confirmed the activation of mitochondria mediated apoptotic pathway^35^. Protein of Bad and Bax interacted with and decreased the levels of Bcl-2 family, which indicates that eupatorin led to a shift from anti-apoptosis to pro-apoptosis by altering the functions of the proteins in the Bcl-2 family^35^. Bax protein oligomerized at the outer mitochondrial membrane and promoted the opening of the mitochondria permeability transition pore, leading to the release of cytochrome c and SMAC/Diablo into the cytosol^36^. The activation of SMAC/Diablo and cytochrome c helped to generate the final step of apoptosis by binding the anti-apoptotic protein of Bcl-2 family and allowing the initiation of downstream caspase such as caspase 3/7, 8 and 9^37^. The release of cytochrome c triggered the activation of caspase 9 through the formation of apoptosome^21,38^. The activation of the initiator caspase 9 caused the cleavage of effectors caspase 3/7 which then activated DNase and caused DNA fragmentation in the nucleus to form necrotic cells^21,39^. Apoptosis induced by eupatorin was mainly activated through intrinsic pathway due to higher fold of activated caspase 9 compared to caspase 8.

In Western blot analysis, growth inhibition of MCF-7 and MDA-MB-231 cells by eupatorin incubation was demonstrated to occur due to inactivation of Akt signaling pathway. Akt signaling pathway was reported to play a pivotal role in tumorigenesis because it affected the growth and survival of cancer cells^40^. This pathway was activated in a variety of cancers, which mediated secretion of VEGF and induction of MMP-9, and played a crucial role in regulation of breast cancer cell growth^21,41^. In addition, Akt pathway promotes cells survival through inhibition of apoptosis via Akt phosphorylation. It was proposed by Wróbel & Gregoraszczuk^8^ that Akt pathway promotes cell survival through distinct pathway of phosphorylation of the Bad component of the Bad/Bcl-2 family complex.

In cell cycle/checkpoint, eupatorin altered the survival-related protein, including Rb-raf-1 interaction, Chk1, Chk2 and Cdc2 in the treated MCF-7 and MDA-MB-231 cells. Level of Rb-raf-1 interaction, Chk1 and Cdc2 expression ensures the cells survival, which relates to angiogenesis induction while Chk2 is a tumour suppressor and stimulated in response to DNA damage and replication blockage that occurred within the cancerous cells due to proliferation inhibition^42,43^. Chk1 is the major cell-cycle checkpoint kinase mediating S- and G2-arrest in response to various genotoxic stresses^42^, which prevents the initiation of mitosis^37^. Previous study reported that Chk1 inhibition leads to major decrease in cell viability in triple negative breast cancer cell lines^42^. Hence, depletion of Chk1 protein expression induces a marked reduction of cell viability and led to mitotic catastrophe in cancer cells^42^.

Checkpoint network gene is commonly stimulated, resulting in DNA damage in order to stop the cell cycle and concurrently starts the DNA repair process^42^. Cdc2 protein is involved in maintaining the survival of cancer cells. Disruption of Cdc2 caused cell cycle arrest in the G2/M phase^37^. In contrast, Chk2 is a tumour suppressor and is stimulated to respond to DNA damage and replication blockage that occurs within the cancerous cells. Hence, inhibition of Chk1 and Cdc2 signal could delay cancer cells proliferation and concurrently stimulate Chk2 protein. These results propose that eupatorin may inhibit cells proliferation in MDA-MB-231 and MCF-7 cells through the depletion of Cdc2 and Chk1 protein levels respectively, followed by the activation of Chk2 protein^42,44^. The induction of cell cycle arrest at a specific checkpoint, and thereby inducing apoptosis, is a common mechanism for the cytotoxic effects of anticancer drugs^21^.

Comparing the effect of eupatorin in MCF-7 and MDA-MB-231, the same IC_50_ value were obtained from both cell lines after 48 hours of treatment. However, some different results were observed in the regulation of Akt and cell cycle pathways analysis. This effect may be a result of the predominant of CYP1A1 in MCF-7 and CYP1B1 MDA-MB-231^17,45^. CYP1A1 was reported as a strong metabolizer of eupatorin to flavone cirsiliol^9,16^, which may be the predominant metabolites that contributed to the cytotoxicity in MCF-7 cells. On the other hand, MDA-MB-231 that was predominantly with CYP1B1, was reported as a slow metabolizer of eupatorin, still observed with better cytotoxic effect at 72 hours and even slightly higher degree regulation of the Akt and cell cycle pathway that contributed to anti-invasion and anti-angiogentic effect of eupatorin. This result provides the clue that unmetabolized eupatorin targeting estrogen negative breast cancer cells show potential advantageous targeting characteristics of invasive breast cancer.

In conclusion, the efficacy of eupatorin as an agent that selectively inhibits proliferation of human breast cancer cells *in vitro* was remarkable. Eupatorin demonstrated the potential anti-breast cancer effect of a flavonoid compound through inhibition of proliferation, invasion, migration and angiogenesis of breast cancer cells *in vitro*. In addition, eupatorin down-regulated the pro-angiogenic genes and upregulated the pro-apoptotic genes in both MDA-MB-231 and MCF-7 cells through inhibiting the Akt and cell cycle pathways. These findings, particularly on the high selectivity on breast cancer cells and mechanism of action, may lead to further development of a potential flavone-based anticancer drug. Although most flavonoids are known to easily penetrate *in vitro* cultured cells^16^, their bioavailability *in vivo* usually decreases due to the capability of enzymes such as in the small intestine and in the liver metabolizing the bioactive compounds^15^. In addition, as CYP1A2 has been associated with hepatotoxicity and chemoresistant due to its effect in metabolizing various drugs including eupatorin^17,46^, future studies on *in vivo* efficacy, pharmacokinetic and toxicity of eupatorin are required.

## Materials and Methods

### Cell lines and culture conditions

The MCF-7 cells were cultured in RPMI-1640 (Sigma, USA) while MDA-MB-231 was maintained in Dulbecco’s modified eagle medium (Sigma, USA). The MCF-10a cells were cultured in the mixture medium of Ham’s F-12 (Sigma, USA) and Dulbecco’s modified eagle medium supplemented with 20 ng/mL epidermal growth factor (EGF), 10 μg/mL insulin, and 250 ng/μL hydrocortisone. All media were added with 10% (v/v) heat-inactivated foetal bovine serum (FBS) (PAA, Austria), penicillin (100 I.U/mL) and streptomycin (100 ng/mL) (PAA, Austria). The cells were cultured at 37 °C in a 90% humidified incubator with 5% CO_2_. When the cells were 80% confluent, they were sub-cultured to a fresh media.

### Materials

Eupatorin and doxorubicin (Sigma-Aldrich, USA) were dissolved in DMSO (Sigma, USA) to prepare a master stock solution at a concentration of 1 mg/mL and stored in −20 °C before use. The working solution was freshly prepared in the complete culture medium.

### Cytotoxicity assay

The cells were seeded at a concentration of 0.8 × 10^5^ cells/mL into a 96-well plate. Following overnight adherence, the cells were incubated with the medium alone or with a two-fold serial dilution of eupatorin starting with the highest concentration at 20 μg/mL for 24, 48 and 72 h respectively. Doxorubin tested for 48 h of incubation was served as positive control. Then, 20 μL MTT solution was added to each well and mixed. After 4 h, the supernatants were removed and 100 μL DMSO was added to each well to dissolve the precipitate. The cells viability was estimated by measuring absorbance at 570 nm using a Quant ELISA plate reader (Bio-tek Instruments, USA). The cell viability percentage was calculated based on the absorbance ratio between cell culture treated with eupatorin and the untreated control multiplied by 100 represents cell viability (percentage of control, %).

Selectivity Index (SI) was calculated as the ratio of cytotoxicity (IC_50_) on normal cells (MCF-10a) to cancer cells (MCF-7 and MDA-MB-231). The SI value higher than 3 suggested that the cytotoxic effect of eupatorin was selective towards MCF-7 cells and MDA-MB-231^21,47^.

### Treatment of MCF-7 and MDA-MB-231 cells with eupatorin

MCF-7 and MDA-MB-231 cells were seeded in a 6-well plate with the amount of 2.0 × 10^5^ cells/well and allowed to attach for 24 h. Then, the cells were incubated with the medium alone or with eupatorin at IC_50_ value for 48 h, which were obtained from MTT assay. At 24, 48 and 72 h of incubation time, the cells were detached with Tryple-EDTA (Gibco, UK), washed with PBS and spun down by centrifugation (2000 rpm, 5 minutes) to collect the pellet, prior to the corresponding assay.

### Morphological assessment

#### Light microscope view

For all untreated and treated MCF-7 and MDA-MB-231 cells, the images were viewed at three time points (24, 48 and 72 h) and the images were captured using a light microscope (Nikon, Japan).

#### Morphological *assessment* using SEM

The untreated and treated cells were cultured on a glass slip for 24, 48 and 72 h respectively, fixed in 2.5% glutaradehyde, and followed by fixing in 1% osmium tetroxide, washed with 0.1 M sodium cacodylate buffer, dehydrated with acetone, and followed by drying in critical point dryer (BAL-TEC CPD 030, CA). The samples was mounted to remove charging and sputtering using sputter coater (BAL-TEC SCD 005, CA). Morphological changes of cancer cells were viewed using Variable Pressure Scanning Electron Microscopy (VP-SEM, USA) at 200x and 1000x magnification.

### Scratched assay

A scratched area was created using a sterile 200 μL pipette tip on 90% confluence of serum starved cells. Then, the cells were incubated in complete growth medium (10% FBS) in the absence or presence of eupatorin (5 μg/mL) for 24 hours. Cells migrated into the wound surface were determined under microscope at various time points. The ratio of cell migration was calculated as the percentage of the remaining cell-free area compared with the area of the initial scratched area^48,49^.

### Transwell migration and invasion assay

Cell migration and invasion were assayed using Transwell (BD Biosciences, USA) with PET track-etched membranes. In brief, 5 × 10^3^ serum-starved cells of MDA-MB-231 were resuspended in serum free medium and transferred into the upper chamber of each Transwell. For invasion assay, the upper compartment of Transwell chamber was coated with matrigel (630 μL) which was diluted in serum-free media in 1:3 ratio before the addition of serum-starved cells. Lower chambers contained fresh medium with the presence or absence of 5 μg/mL eupatorin as chemo-attractant. After left overnight, the cells remaining at the upper surface of the membrane were removed using a swab, whereas those cells that had migrated or invaded to the lower membrane surface were fixed with 100% methanol (1 h) and stained with crystal violet 0.5% (2 h). The number of MDA-MB-231 cells migrated and invaded through the filter was photographed and counted using a microscope (Nikon, Japan) at a magnification of 200x.

### Annexin-FITC/propidium iodide double staining

Apoptosis assessment was conducted using flow cytometry to determine the phosphatidyl serine exposed apoptotic cells by Annexin V–FITC and propidium iodide (PI) double staining. The experiment was carried out according to the Life Technologies Apoptosis Assay protocol and the samples were analysed by flow cytometry within 1 hour using the FACSCalibur (Becton Dickinson, CA) in triplicate. Dot-plot graphs were used to illustrate the viable cells (the lower left quadrant), early-phase apoptotic cells (the lower right quadrant), late-phase apoptotic or dead cells (the upper right quadrant) and the necrotic cells (the upper left quadrant).

### Cell cycle analysis using flow cytometer

The cell cycle analysis was carried out according to the protocol in CycleTEST^TM^ PLUS DNA Reagent Kit, and data acquisition and analysis were performed using FACSCalibur (Becton Dickinson, CA).

### Mouse aorta ring assay

An ethical approval was applied for and approved by Institutional Animal Care and Use Committee (IACUC), Universiti Putra Malaysia (UPM), Serdang, Selangor (Ethical no: R009/2015). All animal procedures were performed according to a protocol approved by the IACUC of Universiti Putra Malaysia. A matrigel (150 μL) was allowed to solidify in 48-well plate. Then, mouse aortic endothelial cells were isolated from the aorta of Balb/c mice, using protocol described by Masson *et al*.^50^ with slight modifications. Briefly, the fat and connective tissues were cleared in PBS-Pennstrep (1%) and cut into 1 mm length. The tissues were placed on the matrigel surface and sandwiched with another 150 μL matrigel. Next, 1.5 mL of eupatorin diluted in complete RPMI-1640 media was transferred into the well and incubated at 37 °C (5% CO_2_/95% air). After 10 days, the tissues were viewed under a microscope (Nikon, Japan) and the emergent angiogenic sprouts microvessels from mouse aortas were observed in culture respectively^51^.

### Real-time PCR (qPCR) analysis

cDNA was prepared from MCF-7 and MDA-MB-231 cells. qPCR was performed using CFX96 Real-time system (BIO-RAD, USA) to quantify the relative mRNA expression levels of selected genes. Primers sequences for all genes were acquired from Natural Centre of Biotechnology Information (NCBI) database (National Library of Medicine, US) and was synthesized by Integrated DNA Technologies (IDT, USA) as presented in Supplementary Table 1. Expression of targeted genes was determined using KAPA SYBR® FAST qPCR Kit Master Mix (2x) Universal (KAPA Biosystems, USA). Thermal cycling was initiated at 95 °C for 2 minutes, followed by 40 cycles consisting of denaturation at 95 °C for 10 seconds and combined annealing/extension steps at 60 °C for 25 second. A melting curve analysis was performed by gradually heating the samples from 70 °C to 95 °C with 0.5 °C increment per second while the fluorescence was measured continuously. The standard curve that possessed efficiency (E) ≥ 90% with linear regression (R^2^) ≥ 0.980 was accepted for genes quantification. All data were normalized to the expression of the reference genes using the Bio-Rad CFX manager (version 3.1) software.

### Western blot analysis

All antibodies used were purchased from QIAGEN Biotechnology Malaysia Sdn Bhd (Cell Signaling Technology, USA). Protein was extracted from cell lines using RIPA lysis buffer with a proteinase inhibitor. The protein lysates were separated by using 12.5% sodium dodecyl sulfate polyacrylamide gel electrophoresis (SDS-PAGE, 12.5%) and soaked in Transfer/Towbin buffer for 15 minutes. After separation, proteins were transferred onto a nitrocellulose membrane, blocked with 5% BSA in a TBS/Tween and then probed with primary antibodies at 4 °C, overnight. Then, the membrane was washed three times with TBST and incubated with the species appropriate HRP-conjugated secondary antibody and anti-biotin, HRP-linked Antibody (at 1:1000–1:3000) to detect biotinylated protein markers. Then, the membrane was washed again with TBST. For protein detection and development, the membrane was incubated in 1x Red Alert prior to visualize a clear protein band.

### Apoptotic proteome profiling

Protein lysate was extracted from MCF-7 and MDA-MB-231 cells and the total protein of cell lysates was determined using Bradford method. In proteome analysis, the targeted protein was identified using Proteome Profiles Human Apoptosis Array kit (R&D systems). The experiment was carried out according to the instruction provided by the manufacturer. The protein arrays on the membranes were scanned using ChemiDoc MP Imaging System (BIO-RAD, USA). The data were analyzed using Image Lab 4.1 software.

### Quantification of caspase 3/7, 8 and 9

The untreated and treated cells of MCF-7 and MDA-MB-231 were stained with 1 µl of Red-IETD-FMK (caspase 8 or 9) or PI (caspase 3/7) 1 h. Then, the pellet was collected and re-suspended in 100 μL wash buffer. The fluorescence intensity was measured at excitation/emission (Ex/Em) = 540/570 nm for caspase 8 and 9 while the fluorescence intensity at Ex/Em = 548/631 was measured for caspase 3 using a fluorescence spectrophotometer (Hitachi, Japan).

### Statistical analysis

Data were obtained from three separate experiments and presented as the mean ± standard deviation of the mean. T test was employed for comparison among multiple groups using GraphPad Prism 6 software. A value of p < 0.05 was considered as statistically significant.

## Supplementary information

Supplementary Figure 1

## References

[1] Adebayo BO (2015). Ovatodiolide sensitizes aggressive breast cancer cells to doxorubicin anticancer activity, eliminates their cancer stem cell-like phenotype, and reduces doxorubicin-associated toxicity. Cancer Lett..

[2] Ahmad I (2015). & Shagufta. Recent developments in steroidal and nonsteroidal aromatase inhibitors for the chemoprevention of estrogen-dependent breast cancer. Eur. J. Med. Chem..

[3] La porta E, Welsh J (2014). Modelling vitamin D in triple negative/basal likebreast cancer-Review. J. Steroid Biochem. Mol. Bio..

[4] Androutsopoulos VP, Tsatsakis AM (2013). Benzo[a]pyrene sensitizes MCF7 breast cancer cells to induction of G1 arrest by the natural flavonoid eupatorin-5-methyl ether, via activation of cell signaling proteins and CYP1-mediated metabolism. Toxicol. Lett..

[5] Park EJ (2009). Down-regulation of c-Src/EGFR-mediated signaling activation is involved in the honokiol-induced cell cycle arrest and apoptosis in MDA-MB-231 human breast cancer cells. Cancer Lett..

[6] Yam MF (2012). Simple isocratic HPLC method for the simultaneous determination of sinensetin, eupatorin, and 3′-hydroxy-5,6,7,4′-tetramethoxyflavone in *Orthosiphon stamineus* extracts. J. Acupunct. Meridian. Stud..

[7] Kim YM (2014). CKD712, a synthetic isoquinoline alkaloid, enhances the anti-cancer effects of paclitaxel in MDA-MB-231 cells through regulation of PTEN. Life Sci..

[8] Wróbel AM, Gregoraszczuk EŁ (2015). Action of methyl-, propyl- and butylparaben on GPR30 gene and protein expression, cAMP levels and activation of ERK1/2 and PI3K/Akt signaling pathways in MCF-7 breast cancer cells and MCF-10a non-transformed breast epithelial cells. Toxicol. Lett..

[9] Androutsopoulos V, Arroo RRJ, Hall JF, Surichan S, Potter G (2008). Antiproliferative and cytostatic effects of the natural product eupatorin on MDA-MB-468 human breast cancer cells due to CYP1-mediated metabolism. Breast Cancer Res..

[10] Salmela AL (2012). The flavonoid eupatorin inactivates the mitotic checkpoint leading to polyploidy and apoptosis. Exp. Cell Res..

[11] Dolečková I (2012). Antiproliferative and antiangiogenic effects of flavone eupatorin, an active constituent of chloroform extract of *Orthosiphon stamineus* leaves. Fitoterapia.

[12] Zheng N (2015). ERα down-regulation plays a key role in silibinin-induced autophagy and apoptosis in human breast cancer MCF-7 cells. J. Pharmacol. Sci..

[13] Levenson A, Jordan V (1997). MCF-7: the first hormone-responsive breast cancer cell line. Cancer Res..

[14] Gest C (2013). Rac3 induces a molecular pathway triggering breast cancer cell aggressiveness: differences in MDA-MB-231 and MCF-7 breast cancer cell lines. BMC Cancer.

[15] Walle T (2007). Methoxylated flavones, a superior cancer chemopreventive flavonoid subclass?. Seminars Cancer Bio..

[16] Androutsopoulos VP, Ruparelia K, Arroo RRJ, Tsatsakis AM, Spandidos DA (2009). CYP1-mediated antiproliferative activity of dietary flavonoids in MDA-MB-468 breast cancer cells. Toxicol.

[17] Androutsopoulos VP, Papakyriakou A, Vourloumis D, Spandidos DA (2011). Comparative CYP1A1 and CYP1B1 substrate and inhibitor profile of dietary flavonoid. Bioorganic & Med Chem.

[18] Nebert DW, Wikvall K, Miller WL (2013). Human cytochromes P450 in health and disease. Philos Trans R Soc Lond B Biol Sci.

[19] Spink DC (1998). Differential expression of CYP1A1 and CYP1B1 in human breast epithelial cells and breast tumor cells. Carcinogenesis.

[20] Pan Y (2014). *In vitro* effect of important herbal active constituents on human cytochrome P450 1A2 (CYP1A2) activity. Phytomedicine.

[21] Li LK, Rola A, Kaid FA, Manaf A, Alabsi AM (2016). Goniothalamin induces cell cycle arrest and apoptosis in H400 human oral squamous cell carcinoma: A caspase-dependent mitochondrial-mediated pathway with downregulation of NF- kb. Arch. Oral Biol..

[22] Havlík M, Dolensky B (2015). Caffeine – hydrazones as anticancer agents with pronounced selectivity toward T-lymphoblastic leukaemia cells. Bioorg. Chem..

[23] Galindo-Hernandez O, Serna-Marquez N, Castillo-Sanchez R, Salazar EP (2014). Extracellular vesicles fromMDA-MB-231 breast cancer cells stimulated with linoleic acid promote an EMT-like process in MCF-10a cells. Prostaglandins, Leukotrienes & Essential Fatty Acids.

[24] Yu JM (2015). BCL6 induces EMT by promoting the ZEB1-mediated transcription repression of E-cadherin in breast cancer cells. Cancer. Lett.

[25] Adler EP, Lemken C, Katchen NS, Kurt R (2003). A dual role for tumor-derived chemokine RANTES (CCL5). Immunol. Lett..

[26] Karroum A (2012). Tubular network formation by adriamycin-resistant MCF-7 breast cancer cells is closely linked to MMP-9 and VEGFR-2/VEGFR-3 over-expressions. Eur. J. Pharmacol..

[27] Kumar AHS (2014). Intravascular cell delivery device for therapeutic VEGF-induced angiogenesis in chronic vascular occlusion. Biomaterials.

[28] Medeiros PJ, Jackson DN (2013). Neuropeptide Y Y5-receptor activation on breast cancer cells acts as a paracrine system that stimulates VEGF expression and secretion to promote angiogenesis. Peptides.

[29] Terwisscha van Scheltinga, A. G. T., *et al*. Visualising dual downregulation of insulin-like growth factor receptor-1 and vascular endothelial growth factor-A by heat shock protein 90 inhibition effect in triple negative breast cancer. *Eur. J. Cancer* **50**, 2508–2516 (2014). 10.1016/j.ejca.2014.06.008 25027745

[30] Saraswati S, Agrawal SS (2012). Antiangiogenic and cytotoxic activity of boswellic acid on breast cancer MCF-7 cells. Biomed. Prev. Nutr..

[31] Bellacen K, Lewis EC (2009). Aortic ring assay. J. Visualized Exp.:JoVE.

[32] Nicosia RF, Zorzi P, Ligresti G, Morishita A, Aplin AC (2012). Paracrine regulation of angiogenesis by different cell types in the aorta ring model. Intern. J. Dev. Bio..

[33] Wang J (2015). CIAPIN1 targets Na^+^/H^+^ exchanger 1 to mediate MDA-MB-231 cells’ metastasis through regulation of MMPs via ERK1/2 signaling pathway. Exp. Cell Res..

[34] Zhang Y (2012). Dryofragin, a phloroglucinol derivative, induces apoptosis in human breast cancer MCF-7 cells through ROS-mediated mitochondrial pathway. Chemico-Biol. Interac..

[35] Atmaca H, Bozkurt E, Uzunoglu S, Uslu R, Karaca B (2013). A diverse induction of apoptosis by trabectedin in MCF-7 (HER2−/ER+) and MDA-MB-453 (HER2+/ER−) breast cancer cells. Toxicol. Lett..

[36] Ma ZY (2012). Activities of a novel Schiff Base copper(II) complex on growth inhibition and apoptosis induction toward MCF-7 human breast cancer cells via mitochondrial pathway. J. Inorg. Biochem..

[37] Lee EJ, Oh SY, Sung MK (2012). Luteolin exerts anti-tumor activity through the suppression of epidermal growth factor receptor-mediated pathway in MDA-MB-231 ER-negative breast cancer cells. Food Chem. Toxicol.

[38] Lin KL (2010). Down-regulation of the JAK2/PI3K-mediated signaling activation is involved in Taiwan cobra cardiotoxin III-induced apoptosis of human breast MDA-MB-231 cancer cells. Toxicon.

[39] Shoja MH, Reddy ND, Nayak PG, Srinivasan KK, Rao CM (2015). Glycosmis pentaphylla (Retz.) DC arrests cell cycle and induces apoptosis via caspase-3/7 activation in breast cancer cells. J. Ethnopharmacol..

[40] Scodelaro Bilbao P, Boland R (2013). Extracellular ATP regulates FoxO family of transcription factors and cell cycle progression through PI3K/Akt in MCF-7cells. Biochimica et Biophys. Acta - General Subjects..

[41] Lin KL, Tsai PC, Hsieh CY, Chang LS, Lin SR (2011). Antimetastatic effect and mechanism of ovatodiolide in MDA-MB-231 human breast cancer cells. Chemico-Bio Intern..

[42] Albiges L (2014). Chk1 as a new therapeutic target in triple-negative breast cancer. The Breast.

[43] Xu C, Wang Q, Feng X, Bo Y (2014). Effect of evodiagenine mediates photocytotoxicity on human breast cancer cells MDA-MB-231 through inhibition of PI3K/AKT/mTOR and activation of p38 pathways. Fitoterapia.

[44] Guo M, Wang M, Deng H, Zhang X, Wang ZY (2013). A novel anticancer agent Broussoflavonol B downregulates estrogen receptor (ER)-α36 expression and inhibits growth of ER-negative breast cancer MDA-MB-231 cells. Eur. J. Pharmacol..

[45] Szaefer H, Licznerska B, Krajka-Kuzniak V, Bartoszek A, Baer-Dubowska W (2012). Modulation of CYP1A1, CYP1A2 and CYP1B1 expression by cabbage juices and indoles in human breast cell lines. Nutr Cancer.

[46] Chen Y (2017). The expression, induction and pharmacological activity of CYP1A2 are post-transcriptionally regulated by microRNA has-miR-132-5p. Biochem Pharmacol.

[47] Wu Z, Shah A, Patel N, Yuan X (2010). Development of methotrexate proline prodrug to overcome resistance by MDA-MB-231 cells. Bioorg. Med. Chem. Lett..

[48] Chun J, Kim YS (2013). Platycodin D inhibits migration, invasion, and growth of MDA-MB-231 human breast cancer cells via suppression of EGFR-mediated Akt and MAPK pathways. Chemico-Biol. Interac..

[49] Okamoto T (2014). Endothelial connexin32 enhances angiogenesis by positively regulating tube formation and cell migration. Exp. Cell Res..

[50] Masson V (2002). Mouse aortic ring assay: A new approach of the molecular genetics of angiogenesis. Biol. Proced. Online.

[51] De Rossi G, Scotland R, Whiteford J (2013). Critical factors in measuring angiogenesis using the aortic ring model. J. Genet. Synd. Gene Ther..

