# Supplementary material for: Cytotoxicity of eupatorin in MCF-7 and MDA-MB-231 human breast cancer cells via cell cycle arrest, anti-angiogenesis and induction of apoptosis
Source: Sci Rep. 2019 Feb 6;9:1514. doi: 10.1038/s41598-018-37796-w (PMC6365513; doi:10.1038/s41598-018-37796-w)

# Cytotoxicity of eupatorin in MCF-7 and MDA-MB-231 human breast cancer cells via cell cycle arrest, anti-angiogenesis and induction of apoptosis

Nursyamirah Abd Razak[1]., Nadiah Abu[2], Wan Yong Ho[3], Nur Rizi Zamberi[1], Sheau Wei Tan[1], Noorjahan Banu Alitheen[4], Kamariah Long[5], Swee Keong Yeap[1,6]*

[1]*Laborotary of Vaccines and Immunotherapeutics, Institute of Bioscience, Universiti Putra Malaysia, Serdang 43400, Selangor, Malaysia.*

[2]*UKM Molecular Biology Institute (UMBI), UKM Medical Centre, Jalan Yaa'cob Latiff, Bandar Tun Razak, Cheras 56000, Kuala Lumpur, Malaysia*

[3]*School of Biomedical Sciences, The University of Nottingham Malaysia Campus, Jalan Broga, Semenyih 43500, Selangor, Malaysia*

[4]*Department of Cell and Molecular Biology, Faculty of Biotechnology and Biomolecular Sciences, Universiti Putra Malaysia, Serdang 43400, Selangor, Malaysia. E-mail: Nanoorjahan@upm.edu.my*

[5]*Malaysian Agricultural Research and Development Institute (MARDI), Serdang 43400, Selangor, Malaysia.*

[6]*China-ASEAN College of Marine Sciences, Xiamen University Malaysia, Jalan Sunsuria, Bandar Sunsuria, Sepang 43900, Selangor, Malaysia*

* Correspondence should be addressed to Swee Keong Yeap

Email: skyeap2005@gmail.com; Tel: +60-3-88006901

Supplementary Table 1 Primers for real-time PCR (qPCR)

| Primers | Accession number | Primer sequence | |
|---|---|---|---|
| | | Forward (5’-3’) | Reverse (5’-3) |
| **Bax** | NM_001291428 | TCTGACGGCAACTTCAACTG | AGTCCAATGTCCAGCCCATG |
| **Bak1** | NM_001188 | CCTGCCCTCTGCTTCTGA | CTGCTGATGGCGGTAAAAA |
| **HIF1A** | NM_001243084 | CCGCTGAAACGCCAAAGCCAC | TTGCGCTTTCAGGGCTTGCG |
| **BCL2L11** | NM_001204106 | CTGCTGGACACACACATACA | GGGCTGAGGAAACAGAGTAAA |
| **VEGFA** | NM_001025366 | CAGCACAACAAATGTGAATGCA | ACACGTCTGCGGATCTTGTACA |
| **GAPDH** | NM_001256799.1 | CACCCACTCCTCCACCTTTG | CCACCACCCTGTTGCTGTAG |
| **β-actin** | NM_001101.3 | TTCTACAATGAGCTGCGTGTG | GGGGTGTTGAAGGTCTCAAA |

**Supplementary Figure 1**. Effect of eupatorin on (A) MCF-10a, (B) MCF-7 and (C) MDA-MB-231 cell cytotoxicity for 24, 48 and 72 hours using MTT assay. Values are expressed as mean±S.D for three independent observations.

Supplementary Figure 1.

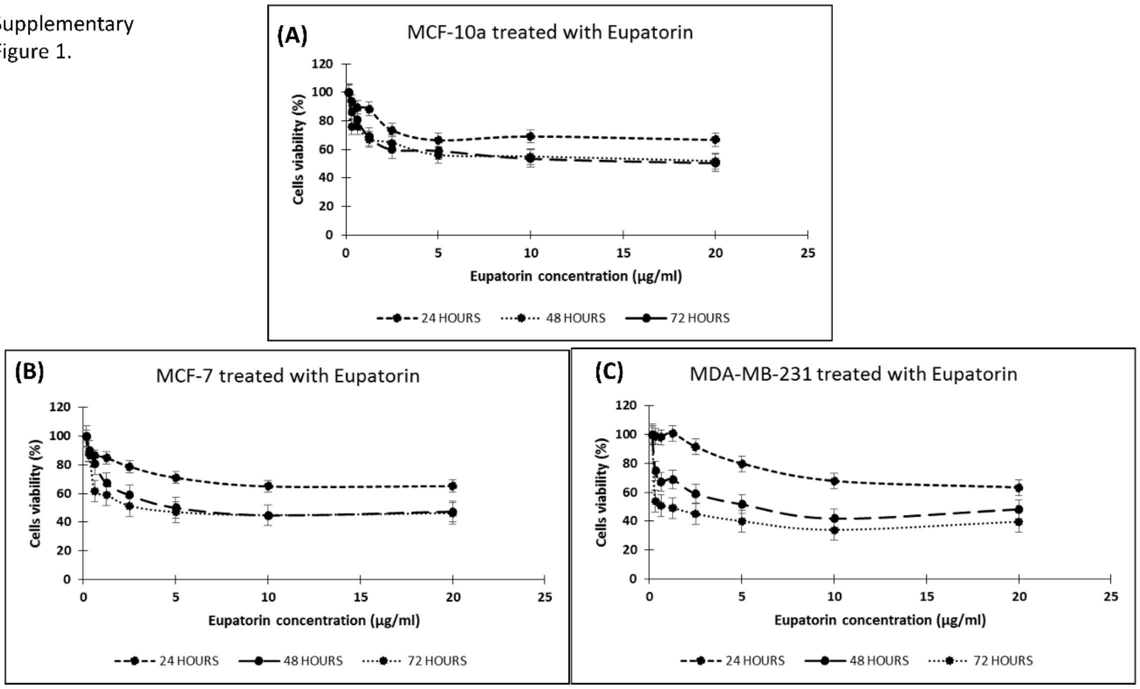

Supplement: Supplementary file 1 — Supplementary Figure 1 [file 41598_2018_37796_MOESM1_ESM.pdf]
